# KENT AND KLEEMEIER AWARD LECTURES

**DOI:** 10.1093/geroni/igz038.2229

**Published:** 2019-11-08

**Authors:** Andrzej Bartke

**Affiliations:** Southern Illinois University School of Medicine, Springfield, Illinois, United States

## Abstract

The Donald P. Kent Award is given annually to a member of the The Gerontological Society of America who best exemplifies the highest standards of professional leadership in gerontology through teaching, service, and interpretation of gerontology to the larger society. The Kent lecture will feature an address by the 2018 recipient, Lewis Lipsitz, MD, Hebrew Senior Life. The Robert W. Kleemeier Award is given annually to a member of The Gerontological Society of America in recognition for outstanding research in the field of gerontology. The Kleemeier lecture will feature an address by the 2018 recipient, Keith Whitfield, PhD, Wayne State University.

